# Iatrogenic Abdominal Pain: A Case Report of a Retained Surgical Item Detected 20 Years After Surgery

**DOI:** 10.7759/cureus.26962

**Published:** 2022-07-18

**Authors:** Inês Nabais, Rita Tinoco Magalhães, Rita Gonçalves Correia, Nuno Saraiva de Melo, Diogo Cruz

**Affiliations:** 1 Internal Medicine, Hospital de Cascais Dr. José de Almeida, Cascais, PRT; 2 Physical Medicine and Rehabilitation, Centro de Medicina de Reabilitação de Alcoitão, Cascais, PRT

**Keywords:** iatrogenic complication, gastrointestinal stromal tumor (gist), gauze piece, foreign bodies, retained surgical item (rsi)

## Abstract

A retained surgical item (RSI) refers to a surgical object (surgical sponges, needles, instruments, device fragments, irrigation sets, guidewires, clips, and rubber materials) accidentally left inside the patient at the end of a surgery or any other procedure. It is considered a *never event* that can have severe consequences for the patient, and that may lead to death. The use of checklists and the implementation of clinical and procedure protocols have attempted to reduce their incidence, but they continue to occur. Most RSI are discovered within three months, with a rare number of cases being diagnosed 3.5 years after the original procedure.

In this report, we discuss the case of a 65-year-old woman who presented with weight loss and B symptoms for a month, a condition resulting from a 20-year RSI, a unique case given the time period between the previous surgery and its diagnosis.

## Introduction

A retained surgical item (RSI), also known as the unintended retention of a foreign object, is a surgical item left inside a patient at the end of surgery and represents a risk to the patient’s well-being. It is usually associated with the need for reoperation, longer hospital stays, or readmission. It is an important cause of infection with a possibility of sepsis or septic shock, mechanical obstruction, and perforation, which can, in turn, lead to the patient’s death [1].

RSI can be characterized according to the type of surgical item left behind, being divided into soft (e.g., sponges, gauze, packing, towels) and hard foreign bodies (e.g., needles, blades, guidewires) [1]. RSI of surgical sponges or gauze are referred to as gossypiboma. This unintentional surgical event is considered a rare occurrence, but the exact incidence is unknown as it is under-reported due to the fear of legal consequences [2].

The operating room is a complex environment with high levels of stress and pressure that accompany technically difficult procedures. Within this setting, there is a high potential for complications and a greater risk of errors that can lead to RSI [3]. Joint Commission reviews identified RSI as the most frequent sentinel event, surpassing other errors such as wrong patient, wrong site, and/or wrong procedure [3].

RSI should always be removed upon diagnosis given the risk that they represent [2]. Most RSI are detected after clinical discharge when symptoms and signs arise, usually in the weeks after the intervention. Nevertheless, some cases can take months to years to be detected as patients remain asymptomatic [3]. We present a case of an RSI with more than 20 years between surgery and the development of symptoms that led to its diagnosis.

## Case presentation

We report a case of a 65-year-old woman, leucodermic, autonomous, retired plumber, with gastric mucocellular adenocarcinoma operated in 1999 (extended partial gastrectomy and epiplonectomy with Billroth II anastomosis with clear surgical margins) and afterward submitted to chemotherapy (unspecified). Close follow-up was carried out in a specialist consultation and, later, by her general practitioner, with a thoracic-abdominal-pelvic (TAP) computed tomography (CT) and endoscopic screening performed every two years, having been apparently free of disease for about 20 years. Regarding family medical records, there was a history of gastric cancer (unspecified) in both the patient's mother and brother.

The patient was admitted to the emergency department with a two-day history of severe pain and swelling of the right lower limb. She had also been displaying, for about a month, symptoms of anorexia, weight loss of approximately 8 kg (more than 10% of her body weight), night sweats, fever, and abdominal pain.

Blood test results showed there was a microcytic and hypochromic anemia with hemoglobin of 6.9 g/dL (confirmed to be iron deficiency anemia, requiring blood transfusion), an elevation of inflammatory parameters with leukocytosis of 15820x10^6^/L, neutrophilia of 12550x10^6^/L, a C-reactive protein of 10.88 mg/dL, an erythrocyte sedimentation rate of 88 mm/h, and slightly elevated D-dimers of 2.55 mg/L. Given her symptoms and complaints, a Doppler ultrasound of the lower limbs was performed and revealed signs of recent medial and lateral gastrocnemius deep vein thrombosis. She was hospitalized with a consumptive condition of unknown etiology, and deep venous thrombosis initially assumed to be paraneoplastic.

**Figure 1:**
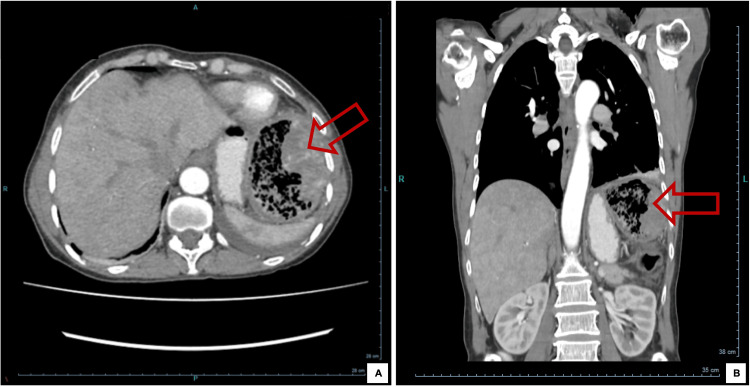
Abdominal CT angiography, axial section (A) and coronal section (B) Figure on mediastinal window showing necrotic mass with a gaseous component and vascularized in the upper left quadrant of the abdomen, subdiaphragmatic, with 9 cm of longest axis, of probable colic origin.

She underwent an upper digestive endoscopy that showed evidence of a stomach operated with permeable efferent and afferent loops without lesions and a colonoscopy that revealed a fistulous orifice in the descending colon, without any other changes. Her TAP CT angiography showed a necrotic mass with a gaseous component and vascularized in the upper left quadrant of the abdomen, subdiaphragmatic, with 9 cm in longest axis, of probable colic origin (Figure 1). It should be noted that pulmonary embolism was excluded. At this stage, considering the personal and family history, clinical presentation, analytical results, and radiological characteristics, the hypotheses of a neoplastic process (namely gastrointestinal stromal tumor (GIST) or lymphoma) and of abscess were raised. An ultrasound-guided percutaneous biopsy was performed, and the resulting anatomopathological study showed fragments of mesenchymal proliferation, consisting of spindle cells, with areas of ischemic necrosis and hemosiderin deposits which favored the hypothesis of GIST. To exclude an infectious process, blood cultures were performed, which were negative, and given the hypothesis of lymphoproliferative disease, immunophenotyping of peripheral blood and medullary blood was performed, but cells of mature lymphoid neoplasia were not detected. Immunofixation revealed a small monoclonal IgG lambda fraction; however, bone marrow aspiration and biopsy showed no changes.

**Figure 2:**
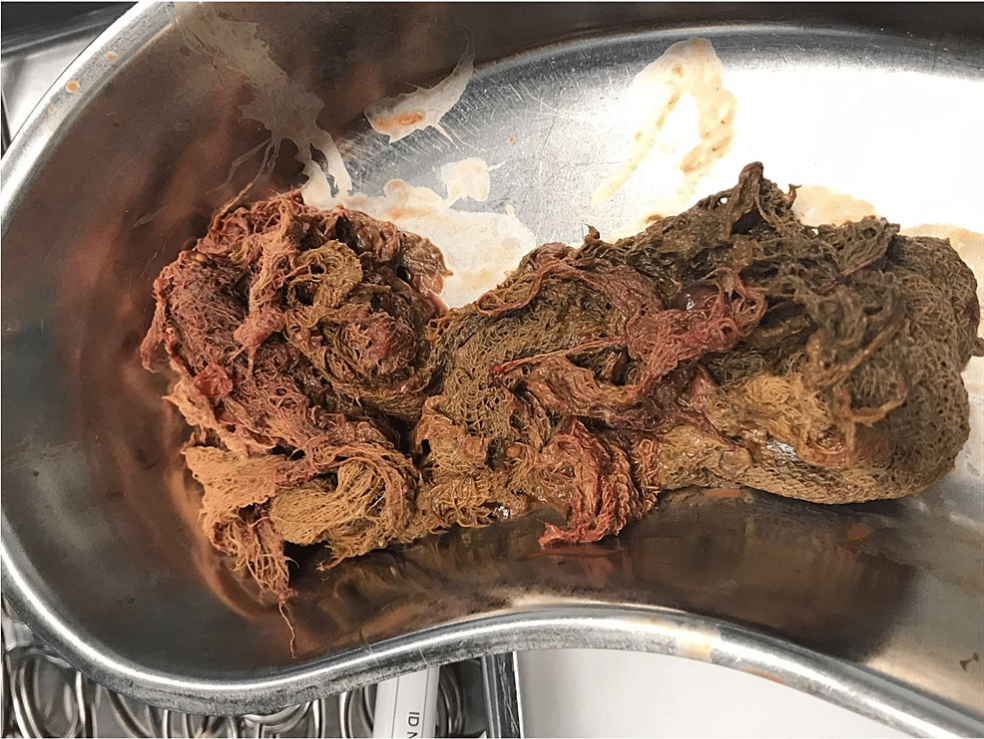
Surgical specimen Postoperative foreign body (gauze) after extraction from the abdominal cavity.

After discussing the case with general surgery, she underwent surgical resection with segmental colectomy, whose macroscopic and histological results did not confirm the results previously obtained in the biopsy. On the other hand, a large gauze was noted (Figure 2). The anatomopathological report described a colonic segment with a granulomatous formation of a foreign body type in the subserosa, in relation to the gauze, with abscess formation and a fistulous path to the mucosa, without dysplasia. The surgical sites were free of injury. Lymph nodes were reactive. Blood cultures and purulent exudate collected intraoperatively isolated *Escherichia coli*, *Streptococcus parasanguinis, *and *Streptococcus salivarius,* so a complete course of 10 days of directed antibiotic therapy was given.

It is important to state that, given the case described, the medical team asked for and reviewed the complementary diagnostic tests carried out over the 20 years of follow-up. They indeed mentioned the presence of a "mass" located in the upper left quadrant, but the patient was unaware of this information because the reports were never opened or analyzed by any other physician.

## Discussion

Abdominal pain accompanied by weight loss suggests, in most cases, inflammatory bowel disease, chronic mesenteric ischemia, gastrointestinal neoplasia, or, eventually, intra-abdominal abscess [4]. Differential diagnosis encompasses several diseases with very different treatments and prognoses, and therefore, the etiological investigation to reach a definitive diagnosis is essential for a correct approach to patients.

In this case, the known past medical history, together with the clinic and the complementary diagnostic tests, suggested a neoplastic process, despite the confirmation later that it was due to the presence of a foreign body. In fact, GIST is the most common mesenchymal tumor, usually located in the stomach (60-70%), followed by the small bowel (20-30%), colon-rectum (5-10%), and finally the esophagus (5%). In spite of that, it only accounts for 2% of all malignant gastric tumors [5]. At the same time, the synchronous occurrence of gastric GIST and gastric cancer has been reported more frequently in recent years [5], and according to a review of 4813 symptomatic GIST patients, the frequency of coexisting malignant tumor was 10.1%, and that of gastric cancer was about 2% [6]. So, considering the history of gastric adenocarcinoma, the reason for admission, and the imaging exam suggestive of GIST, there was a strong likelihood of this possibility in the early stages of the investigation. 

In terms of follow-up and prognosis, the fact that the case was not cancer-related, but a complication of a soft retained foreign body from a previous operation, makes it more favorable for the patient. However, a retained foreign body is considered iatrogenic, with potential consequences of varying severity that could and should have been avoided.

The incidence of cases of surgical material retained in the abdominal cavity is low, in the order of one in every 1000 to 1500 intra-abdominal surgical interventions [7]. Analyzing all types of surgery globally, a retrospective study by Susmallian et al., published in 2022, concluded that the rate of RSI in their series was 0.259/1000 operations, with a probability of 1/3888. They also verified that the number of RSI reports increased annually by an average growth of 34.38% per year, with a total increase of 171.92% during the study period [8]​​​​​​. However, the use of preventive measures made it possible to reduce the number of events. Thus, identifying potential risk factors and implementing clinical and procedure protocols that minimize the probability of inadvertent retention of surgical material is of the utmost importance. There are several factors significantly associated with a higher risk for an RSI event, including blood loss greater than 500 cc, prolonged operative time, more than one sub-procedure, more than one surgical team, unexpected intra-operative findings, lack of surgical counts and incorrect counts, body mass index (BMI) above 35 kg/m^2^, and on how well the members of the surgical team work together (e.g., communication failure, distractibility, and the lack of adaptability). Different strategies have been investigated and should be applied to avoid these events, such as counting systems and intraoperative radiography. Other methodologies (e.g., computer-aided detection, magnetic retrievers, sharp detector, etc.) are still under investigation [9].

Regarding the permanence time of a foreign body, according to Lincourt et al., the mean number of days to the discovery of RSI was 93 days, with the longest discovery time 3.5 years after the initial surgical procedure [10]. We report this case, which describes an event that is rare in itself but which becomes unique as the patient remained completely asymptomatic for 20 years with the presence of a considerably large foreign body in the abdominal cavity.

## Conclusions

Taking into account the low incidence of RSI (although growing in recent years) and the long time that elapsed between surgery and the onset of clinical manifestations (20 years), this case demonstrates the importance of excluding what is common without forgetting what is unlikely. Since they are referred to in the literature as *never events*, RSI must be understood as preventable occurrences, with control measures and technology aiding and allowing for fewer occurrences and helping their eradication.
